# Reducing Alcohol Use Before and After Surgery: Qualitative Study of Two Treatment Approaches

**DOI:** 10.2196/42532

**Published:** 2023-07-26

**Authors:** Lyndsay Chapman, Tom Ren, Jake Solka, Angela R Bazzi, Brian Borsari, Michael J Mello, Anne C Fernandez

**Affiliations:** 1 Department of Psychiatry University of Michigan Medical School Ann Arbor, MI United States; 2 College of Medicine Central Michigan University Mount Pleasant, MI United States; 3 Herbert Wertheim School of Public Health University of California, San Diego San Diego, CA United States; 4 Department of Community Health Sciences Boston University School of Public Health Boston, MA United States; 5 Mental Health Service San Francisco VA Medical Center San Francisco, CA United States; 6 Department of Psychiatry and Behavioral Sciences University of California, San Francisco San Francisco, CA United States; 7 Department of Emergency Medicine Rhode Island Hospital Providence, RI United States; 8 Department of Health Services, Policy and Practice Brown University School of Public Health Providence, RI United States

**Keywords:** alcohol use, brief intervention, surgery, preoperative, alcohol use disorder, alcohol, substance use, substance abuse, postoperative, perioperative, counseling, surgical

## Abstract

**Background:**

High-risk alcohol use is a common preventable risk factor for postoperative complications, admission to intensive care, and longer hospital stays. Short-term abstinence from alcohol use (2 to 4 weeks) prior to surgery is linked to a lower likelihood of postoperative complications.

**Objective:**

The study aimed to explore the acceptability and feasibility of 2 brief counseling approaches to reduce alcohol use in elective surgical patients with high-risk alcohol use in the perioperative period.

**Methods:**

A semistructured interview study was conducted with a group of “high responders” (who reduced alcohol use ≥50% postbaseline) and “low responders” (who reduced alcohol use by ≤25% postbaseline) after their completion of a pilot trial to explore the acceptability and perceived impacts on drinking behaviors of the 2 counseling interventions delivered remotely by phone or video call. Interview transcripts were analyzed using thematic analysis.

**Results:**

In total, 19 participants (10 high responders and 9 low responders) from the parent trial took part in interviews. Three main themes were identified: (1) the intervention content was novel and impactful, (2) the choice of intervention modality enhanced participant engagement in the intervention, and (3) factors external to the interventions also influenced alcohol use.

**Conclusions:**

The findings support the acceptability of both high- and low-intensity brief counseling approaches. Elective surgical patients are interested in receiving alcohol-focused education, and further research is needed to test the effectiveness of these interventions in reducing drinking before and after surgery.

**Trial Registration:**

ClinicalTrials.gov NCT03929562; https://clinicaltrials.gov/ct2/show/NCT03929562

## Introduction

High-risk alcohol use (often defined as 2 or more drinks per day [1], or a score of 5 or more on the Alcohol Use Disorders Identification Test Consumption [AUDIT-C] [2,3] screening tool) is a common preventable risk factor for postoperative complications, admission to intensive care, and longer hospital stays [2-6]. Importantly, alcohol-associated adverse surgical outcomes are not specific to certain surgeries or subpopulations; instead, they are evident across heterogeneous patients and surgery types [3,5]. Short-term abstinence from alcohol use (2 to 4 weeks) prior to surgery is linked to a lower likelihood of postoperative complications [7-13]. Likewise, abstinence of 5 to 6 weeks after surgery is recommended to reduce one’s risk of experiencing complications such as delayed wound healing, infection, and impaired cardiac function [11,14]. In addition, the majority of surgical patients receive an opioid prescription after surgery [15,16], for which concurrent alcohol use is dangerous and even lethal [17,18]. Despite these recommendations and research, elective surgical patients are rarely offered alcohol-focused assessment, education, intervention, or treatment referrals prior to surgery [6,19,20]. For example, one study found only 25% of studies of alcohol use in surgical patients used standardized alcohol assessments, and a qualitative study found surgical care providers often did not have interest in screening or discussing alcohol use with patients.

Currently, the field lacks rigorous research on brief interventions to reduce high-risk alcohol use prior to surgery. Existing preoperative interventions involve pharmacotherapy and frequent in-person visits to promote abstinence, making them most appropriate for individuals at the higher end of the alcohol use and alcohol use disorder spectrum [7,9]. A broader array of treatment options are still needed to address the full spectrum of patients drinking prior to surgery. To address this gap, we developed 2 brief counseling approaches—a low-intensity “brief advice” intervention and a higher-intensity “health coaching” intervention—to reduce preoperative alcohol use. Consistent with the different preferences and implementation needs of patients and clinical staff identified in our formative research, these 2 interventions varied in content, intensity, and modality [21].

In this paper, we describe the acceptability and feasibility of the brief advice and health coaching interventions from qualitative exit interviews with treatment “high responders” (who reduced alcohol use ≥50% postbaseline) and “low responders” (who reduced alcohol use by ≤25% postbaseline). In this way, we sought to gather contextual information and potential reasons for high versus low intervention response, including participants’ perspectives on the impact of specific intervention elements, modalities, and any unanticipated influences on their alcohol use related to surgery preparation or recovery.

## Methods

### Study Design and Sample

This paper reports results of qualitative exit interviews conducted as part of the Alcohol Screening and Preoperative Intervention Research (ASPIRE) study, a randomized pilot trial of 2 preoperative alcohol interventions [22]. ASPIRE recruited 51 participants between July 2019 and February 2021 (with a pause from April to July 2020 due to COVID-19–related reductions in elective surgeries) from a large academic medical center in the Midwestern United States. To be eligible for the parent trial, participants had to meet the following inclusion criteria: (1) be scheduled for elective or semielective surgery in the next 35 to 120 days in select subspecialties (plastic, knee or hip arthroplasty, minimally invasive, endocrine, gynecology, urology, colorectal, or hepatobiliary); (2) receive regional or general anesthesia; (3) be aged 18 to 75 years; (4) have positive or unknown alcohol use in their social history in the electronic health record; and (5) meet criteria for high-risk alcohol use (AUDIT-C score ≥5). Selected surgical types included orthopedic, plastic, urology, gynecological, hepatobiliary, and general surgery. Individuals were excluded if they were undergoing surgeries requiring only local anesthesia, could not read or understand English, or had substantial cognitive impairment or evidence of psychotic symptoms (ie, delusions or hallucinations). Research staff contacted potential participants by phone (call or text). Eligible participants provided written informed consent for all trial procedures including qualitative exit interviews. Randomization into the brief advice or health coaching intervention conditions was stratified based on sex and alcohol screening scores.

### Ethics Approval

The University of Michigan’s Institutional Review Board approved all study protocols (HUM00156743). This is a registered clinical trial (ClinicalTrials.gov NCT03929562).

### Study Interventions

#### Health Coaching

The health coaching intervention consisted of a trained, graduate-level health coach delivering 2 telehealth intervention sessions 4 and 2 weeks prior to surgery. Participants had the choice of taking part in-person (prepandemic only) or through teleconferencing compliant with the US Health Insurance Portability and Accountability Act. Sessions lasted 45 minutes each. Intervention content was based on principles of health coaching, an evidence-based, collaborative care approach that partners patients with coaches who teach strategies to self-manage health behavior. Key features include motivational interviewing, goal setting, and discussion of strategies for alcohol use reduction or cessation [23]. Our health coaching sessions also included a 4-page visual and text-based session guide (emailed to participants in advance) that included health education, personalized feedback, and tailored surgical risk messaging based on the health belief model [24,25]. Health coaching sessions introduced the concept of “prehabilitation,” which involves improving health through behavior change before surgery in order to help improve postoperative outcomes. This message framing emphasized two main health belief factors: (1) the desire to avoid a health problem (ie, surgical complications) and (2) the belief that a given behavior (ie, alcohol cessation) can prevent the problem. Coaching conversations were designed to increase motivation to reduce pre- and postoperative alcohol use. These sessions allowed participants to reflect on and discuss their alcohol use and produce personal perioperative health goals.

#### Brief Advice

The brief advice intervention consisted of one 10-minute phone session led by the same graduate-level health coach 4 weeks prior to surgery. The phone call was accompanied by a 2-page handout (emailed to participants prior to the session) that included information about participants’ reported alcohol use from baseline surveys, educational information about alcohol and surgical health, and advice to stop alcohol use for 4 weeks prior to surgery and for 6 weeks after surgery. Treatment resources and alcohol withdrawal information were included in the handout.

### Quantitative Assessment and Responder Classification

The ASPIRE pilot trial involved 3 quantitative assessment time points: at baseline and 1- and 4-months postbaseline. After the final (4-month) follow-up, treatment response and low response were assessed by calculating percent change in alcohol use at follow-up time points relative to baseline.

#### Alcohol Use

Changes in alcohol use were assessed at baseline and follow-up visits using the AUDIT-C [26] and a web-based version of the Timeline Follow Back (TLFB) [27]. The 3-item AUDIT-C assesses alcohol quantity and frequency with an overall score ranging from 0 to 12 (higher scores reflect higher levels of alcohol use). The TLFB uses a calendar format on which participants self-report the number of standard drinks (approximately 14 g of alcohol) consumed each day to yield measures of average drinks per week and percent of days abstinent. Our assessment time frames included the past 3 months for baseline and final follow-up visits, and past month at the 1-month follow-up visit.

#### Responder Classification

Those who reduced alcohol use more than ≥50% relative to baseline on both the AUDIT-C and TLFB (average drinks per week) were classified as high responders. Those who reduced alcohol use by ≤25% relative to baseline on both the AUDIT-C and TLFB were classified as low responders. Percentage cutoffs were data driven, chosen based on the distribution of change in alcohol use at follow-up. We used 2 measures to classify responder status to ensure the highest level of certainty in our responder and low-responder classification. These 2 measures are highly correlated but also provide slightly different alcohol use data for decision-making.

### Qualitative Data Collection

Following completion of the final follow-up assessments at 4 months for the ASPIRE pilot trial, we invited participants to take part in qualitative exit interviews based on study condition (brief advice and health coaching) and treatment response. Our goal was to enroll approximately at least 25% of the trial sample of N=51. Of the 26 participants invited based on these criteria, 19 agreed and completed exit interviews.

A trained graduate-level interviewer conducted qualitative exit interviews via the internet (through Health Insurance Portability and Accountability Act–compliant teleconferencing) using a semistructured interview guide containing open-ended questions and detailed probes designed to explore how the interventions (or low-intervention factors) may have influenced alcohol and other substance use during the perioperative period (see Multimedia Appendix 1 for the guide). Questions also explored participants’ perspectives on the acceptability (ie, whether the sessions and content were appropriate, appealing, and impactful) and feasibility (ie, whether participation was convenient, clear, and achievable), as well as how comfortable they felt sharing accurate and honest information about substance use with study staff and medical providers. Study staff regularly reviewed transcripts against audio recordings to confirm accuracy and deidentification.

### Qualitative Coding and Data Analysis

We undertook thematic analysis of transcribed data that began with a collaborative codebook development process involving 4 research team members (the principal investigator, study coordinator, research assistant, and a lead qualitative investigator) [28-30]. First, key topics of interest were independently reviewed from the interview guide and several selected transcripts to develop a preliminary list of potential codes and definitions [31]. We met to discuss and refine this preliminary list, which we compiled into a draft codebook. Interviews were audio-recorded and transcribed verbatim by a professional transcription company that omitted potentially identifiable information. The draft codebook was then independently tested using another set of transcripts and the team members met again to discuss and refine the codebook. Through 3 additional rounds of this iterative codebook development and testing process, discrepancies in code application were identified and resolved; codes were merged, added, and removed; and code definitions were revised as necessary until consensus was reached on a final codebook. To validate the final codebook and assess consistency in code application, 2 core coders (the study coordinator and research assistant) double-coded 2 transcripts using MAXQDA (VERBI Software). After assessing and determining that there was a high degree of consistency, we divided up the remaining transcripts between the 2 coders, who then independently coded the remaining transcripts under the supervision of the principal and lead qualitative investigators. We continued holding weekly meetings to review coding progress, discuss emergent themes, and identify and clarify preliminary findings [32]. To delve deeper into emergent themes that were identified during an initial round of coding (described below), midway through data collection, new questions were added on COVID-19 experiences, and intervention changes that emerged in earlier interviews were suggested. For our thematic analysis for this paper, we focused on understanding intervention acceptability and feasibility and potential reasons for intervention response. The transcripts across these domains were then reviewed to identify key themes, which are described below and exemplified using anonymized quotes.

## Results

### Overview

Among 19 participants, 9 (47%) were female, 9 (47%) were from the health coaching intervention condition, 10 (53%) were from the brief advice condition, 9 (47%) were considered high responders, and 10 (53%) were considered low responders (Table 1). From our analysis of interview data, we identified the following three themes: (1) the intervention content was novel and impactful, (2) the choice of intervention modality enhanced participant engagement in the intervention, and (3) factors external to the interventions also influenced alcohol use. These themes are detailed below and summarized in Table 2.

**Table 1:** Qualitative interview participant characteristics at baseline.

		Brief advice (n=10)	Health coaching (n=9)	Total (n=19)
Female sex, n (%)		4 (40)	5 (56)	9 (47)
Identifies as woman, n (%)		4 (40)	4 (44)	8 (42)
Age (years), mean (SD)		47.5 (9.75)	58 (14)	50 (17)
White, n (%)		10 (100)	8 (89)	18 (95)
Non-Hispanic, n (%)		10 (100)	9 (100)	19 (100)
Low responder, n (%)		5 (50)	5 (56)	10 (53)
**Surgical category, n (%)**				
	Orthopedics	2 (20)	5 (56)	7 (37)
	Plastic	4 (40)	3 (33)	7 (37)
	Minimally invasive	2 (20)	N/A^a^	2 (11)
	Other^b^	2 (20)	1 (11)	3 (16)
Drinks per week, mean (SD)		10.4 (12)	9.3 (14.8)	9.6 (14.2)
Average AUDIT-C^c^ score, mean (SD)		5.9 (1.1)	6.1 (1.1)	6.0 (1.1)
Tobacco use, n (%)		1 (10)	5 (56)	6 (32)
Marijuana use, n (%)		2 (20)	4 (44)	6 (32)
Prescription opioid use, n (%)		1 (10)	3 (33)	4 (21)
Other drug use^d^, n (%)		2 (20)	1 (11)	3 (16)

^a^N/A: not applicable.

^b^Urology, endocrine, and gynecology.

^c^AUDIT-C: Alcohol Use Disorders Identification Test–Consumption.

^d^Amphetamines and hallucinogens.

**Table 2:** Summary of qualitative findings by theme and responder status.

Theme	High responders (n=9)	Low responders (n=10)
Intervention content	Perceived information as novel and personally relevant for improving their own surgical health. Changed risk perceptions related to alcohol use and surgery. Opportunity for longer discussions with a nonjudgmental health coach was important (health coaching only).	Information is novel and believable but perceived as only relevant to others who have a more serious “alcohol problem.” The information about alcohol and surgical risk was not shared before past surgeries and therefore inconsistent.
Intervention modality	Participants reported intervention was an acceptable modality and length.	Phone modality limited personal connection, and length was too short to discuss a sensitive topic like alcohol use (brief advice only).
Other influential factors: study assessments	Completing study assessments, which included completing a daily calendar of alcohol use increased awareness and motivation to change.	Completing study assessments increased awareness of alcohol use and motivation but intervention did not (brief advice only).
Other influential factors: intervention language	No thematic findings.	The term intervention caused negative reactions and contributed to participants thinking their alcohol use was not heavy enough for study participation.
Other influential factors: surgery factors	Surgery preparation and recovery influenced choice to reduce or stop drinking for a period of time independent of the intervention content. More intensive surgeries, longer healing time, and continued use of opioid-based pain medications linked to longer alcohol abstinence.	Less intensive surgery and shorter healing time linked to faster return to alcohol use.

### Intervention Content Was Novel and Impactful

Overall, participants found both intervention conditions to be acceptable, of appropriate length, and impactful. Several participants stated that the intervention directly influenced their perioperative alcohol consumption: “It was very enjoyable. It was easy...there hasn’t been a thing that’s made me uncomfortable or that I haven’t agreed with [in] the study.” In general, participants reacted positively to the information presented in both intervention conditions. Participants considered the information regarding the risks of preoperative alcohol use on surgical outcomes to be novel (yet believable) and motivating. By receiving this information, participants described feeling more prepared for surgery and “in control” of their surgical outcomes. As one health coaching intervention high responder explained:

I really did appreciate having that opportunity to say, “This is something that’s a big deal for my body.” Anything I can do to ensure the success of the surgery is something I want to do. Then, I’m talking about [the study] with a lot of people, saying, “I never realized this, but if you are going through something medically, it’s a really good idea to get yourself as ready as possible.”

55 years old, female

Several participants commented on what they learned about the connections between alcohol use and the body’s ability to heal, including alcohol’s impact on the immune system. In considering what “stood out” from the brief advice condition, one high responder explained:

I’ve never had any sort of information beyond just, you know, the day of the surgery, “Don’t eat anything before or after midnight.” You know, the general pre-surgical conditions to get through the day. But this [intervention] made an attempt to look out for my bigger picture health for a longer period of time before and after the surgery.

47 years old, male

Other high responders generally perceived the information shared as part of the intervention as impactful, identifying it as one of the main reasons they reduced their preoperative alcohol use*.* One brief advice high responder (48 years old, male) stated “If I was not a part of the study or aware of the study, I probably would not have reduced [my drinking].” Health coaching participants described how conversations with a nonjudgmental health coach provided them an opportunity to carefully consider changing their alcohol use.

Both interventions presented information about alcohol use in the preoperative period that some participants found helped them make a “connection” between alcohol use and health, ultimately changing their personal risk perceptions. Participants found the concept of prehabilitation, or optimizing health *prior to* surgery, to be particularly impactful because the health coach encouraged them to view themselves as important members of their surgical teams who could enhance their own health, as opposed to passively receiving surgery. As such, they felt an improved sense of control over their surgical outcomes. As one health coaching high responder explained:

Participation gave me more control over the anxiety having to do with the surgery because I’m a person who likes to control things, and it just gave me a sense of control over one more aspect of the surgery that I could do the best I could do to have success.

55 years old, female

Among low responders in both intervention conditions, there was a sense that the information presented was true but did not apply to them personally. This was particularly true for participants on the lower end of the AUDIT-C eligibility assessment, who did not view their drinking habits as risky. These individuals felt they had been included in the study erroneously, with one health coaching low responder (71 years old, female) saying, “it just didn’t feel like a good fit for me.” A brief advice low responder (47 years old, male) further elaborated that he thought the content was “focused on people that drink a lot all the time...while I don’t have a problem having a beer or two on the weekends, it’s really not an issue for me.”

Some participants who had undergone major surgeries in the past described never receiving information about alcohol use and related surgical risks. Accordingly, some participants mentioned feeling surprised that their doctors had not communicated this kind of information to them in the past. One brief advice low responder (22 years old, male) explained that he would have appreciated receiving better information from his surgical team given the significance of the topic, saying, “I’ve had two surgeries in the past on my knee...these issues literally never came up. I was never briefed on what complications might arise around alcohol or tobacco use, so that was new.”

### The Choice of Intervention Modality Enhanced Participant Engagement in the Intervention

By design, intervention modality differed between conditions. While the brief advice condition was only delivered by phone, health coaching participants were given a choice between phone or face-to-face sessions (by videoconferencing or, prior to COVID-19, in-person). Overall, it appeared that having some choice in intervention modality increased participants’ satisfaction; while nearly all health coaching participants were satisfied with their modality of choice, brief advice participants, who had no choice, indicated mixed experiences and dissatisfaction with phone participation.

Some health coaching participants who chose face-to-face intervention delivery expressed high satisfaction with this modality, explaining that they felt more engaged with the information presented, took intervention recommendations more seriously, and were more open and honest because it felt more “personal.” As one health coaching high responder (58 years old, male) explained, “You’re talking to somebody, a human, and it just makes you want to participate and follow the rules, so [there’s] more accountability.” Most face-to-face sessions involved videoconferencing, which participants could schedule around their work and personal lives; several noted that this flexibility enabled them to save time and energy compared with traveling to in-person sessions. They were also able to participate in a setting of their choice, which in addition to being convenient, was more comfortable. Instances of dissatisfaction included one participant stating the sessions were “a bit too long” and another participant who would have preferred in-person sessions, but this was impossible due to COVID-19 pandemic restrictions at that time.

In contrast, brief advice participants’ experiences with phone sessions were more mixed, possibly because they lacked any choice in intervention modality. While some participants discussed specific advantages of phone sessions (eg, greater ease of use and convenience over videoconferencing or traveling to an in-person appointment), others felt they would have been more comfortable face-to-face, with one participant (48 years old, male) saying he would be “a lot more engaged [having] a video in front of me and [knowing] I’m being watched at the same time.” Furthermore, they perceived a poorer connection between themselves and the interventionist, viewing the phone modality as “un-intimate.” As a brief advice low responder explained:

[I was] probably not as comfortable, for whatever reason. It was a phone conversation. I was in a parking lot during work, in between meetings, in my car. I guess the setting wasn’t super great, and now I’m at home [during qualitative interview]. I’m sitting down, I’m not on my phone. Those are all factors.

48 years old, female

Most felt that the length of health coaching sessions (~45 minutes) was appropriate and afforded them sufficient time for discussion without feeling rushed while also showing respect for their time. In contrast, while most participants in the brief advice condition felt that the session length was acceptable, some felt the length was too short to feel comfortable opening up about the sensitive topic of alcohol use.

### Factors External to the Interventions Also Influenced Alcohol Use

There were several factors external to the intervention content that influenced participants’ alcohol consumption, including (1) self-report and tracking of alcohol use for the ASPIRE trial assessments, (2) “intervention” language used in the study, and (3) surgical experiences.

#### Self-Report and Tracking

Assessments for the ASPIRE study included self-reported alcohol consumption using a 3-month retrospective TLFB calendar procedure, which affected participants in 2 ways. First, high responders described that knowing the study team would review their data and goals promoted a sense of “accountability” and not wanting to be caught “breaking the rules” by drinking at higher levels. One health coaching participant (63 years old, male) explained “I think setting the goals and following through, knowing that someone was gonna get it at the end of the time period.... That helped a lot.” Second, self-report and tracking gave some participants an improved awareness of their drinking habits by enabling them to visually recognize behaviors they wanted to change. Some discussed how the calendar helped reveal patterns they had not noticed before, as one brief advice low responder (43 years old, female) explained: “When you look at that calendar, after you fill it out, and go, oh my God, I drank every day for two weeks...there’s no need to be doing it every day. It just seems excessive.” This was especially true for those in the brief advice condition, as study assessment activities, which totaled ~2.5 hours across the duration of the ASPIRE study, were proportionally much longer than the ~10-minute intervention session. As one brief advice high responder (43 years old, female) explained, “I don’t think the intervention content was that impactful. I think it was more of just the [activities], doing the survey, kind of as a journal, that had more of an impact on me.”

#### “Intervention” Language Used in the Study

Some participants reacted negatively to language used during recruitment and study implementation. Study materials initially referred to both conditions as “interventions,” which implied to participants that they had an alcohol-related “problem” significant enough to require help. As one health coaching low responder (71 years old, female) stated, “I mean, it’s hard not to feel defensive when somebody says, ‘Oh, well, you’re being put in an intervention.’” For some participants, this term carried negative connotations leading them to question whether they were a “good fit” for the study, particularly if they did not see themselves as having a “problem.” As one health coaching low responder (71 years old, female) explained:

I was a little taken aback by this sense of, oh my God, you know, an “intervention.” Like, where did that come from, you know? This is not something that’s ever been a particular problem or concern, so it was just a little bit of a surprise.... And all of a sudden, I’m in this category like, “You need an intervention.” I was like, “What? Excuse me? Hello? No, don’t think so.” But anyway, I was mildly taken aback by all that.

#### Surgical Experiences

Most study participants viewed their surgeries as an important life event; some further recognized surgery as dangerous and requiring careful preparation and viewed alcohol consumption as harmful for surgical recovery even before the intervention. For these individuals, reducing alcohol use prior to surgery seemed intuitive, and they believed they would have done so even without the intervention. One brief advice high responder explained:

I know [surgery is] dangerous, just baseline dangerous, so the better health you can be in, the better the surgery will go and the recovery. I guess I was already at that point [of wanting to be healthier for the surgery], so I was—I don’t think [the intervention] had any impact.

47 years old, male

Postoperatively, recovery prevented many participants’ alcohol consumption for at least some period due to not feeling well enough to eat, drink, or ambulate. As one brief advice high responder explained:

Half the time, I didn’t feel like even eatin’ or doin’ anything, so that might not be—it might not be the study that made me do what I did. It might have been the pain level and other factors.

47 years old, male

Others intuitively recognized that drinking could negatively impact their recovery and believed they would have reduced their alcohol consumption even without the intervention. Some participants also decided to abstain from alcohol beyond 6 weeks after surgery to continue improving their health. Others recognized that mixing alcohol and opioid-based medications is dangerous and chose to abstain from alcohol in the postoperative period for such reasons. Those with easier, shorter recovery periods did not view surgery as having a large impact on their decision to drink alcohol one way or another. Low responders began to drink alcohol as soon as they felt better from surgery, even within 6 weeks, as one brief advice participant explained:

[Alcohol] was non-existent the first few days after [the surgery], and then it picked back up shortly after...once I was off [OxyContin], then I was drinking.

43 years old, female

## Discussion

### Principal Findings

This study describes the experiences of participants in a preoperative pilot trial of alcohol-focused interventions of varying intensities and modalities. Participants in our qualitative exit interviews were selected to represent high responders and low responders to help better understand potential effective and ineffective elements of the interventions to inform a future sequential multiple assignment randomized controlled trial, assigning participants to various treatment options based on initial treatment response. Overall, high responders in our pilot trial described intervention content as novel and relevant. The health coaching condition, which involved motivational interviewing and goal setting, provided an opportunity to reflect on alcohol use in an open, nonjudgmental conversation with supporting materials that framed the patients’ roles as active, thereby empowering them to take actions to improve their surgical outcomes. Surgery preparation and recovery appeared to motivate change in alcohol use for participants, with the intervention content helping some participants link their alcohol use to surgical health for the first time. While most participants found both interventions to be novel and motivating, a few believed the link between alcohol use and surgical health was intuitive and that they would have stopped or reduced alcohol use before and after surgery regardless of the intervention. Likewise, longer surgical recovery and prolonged medication use led to longer alcohol cessation for some participants.

Though all participants met “risky” alcohol use eligibility requirements for the study using a validated alcohol use screening and risk identification tool, many did not perceive themselves as drinking enough to put them at risk, especially in the low-responder group. Instead, some of these participants described dismissing the intervention content about alcohol use and surgical risk because they felt it did not apply to them. A modified approach that uses different terminology or alternative discussion points could help address this barrier to change. This could include acknowledging that some participants may be surprised by feedback suggesting their level of alcohol use could be linked to surgical risks and discussing their reactions to this information.

There were also aspects of study participation and surgery that influenced participants’ alcohol use separate from the intervention content. There was evidence of assessment reactivity, particularly for participants in the brief advice condition for whom the baseline and follow-up study assessments represented a much larger proportion of their participation time than the actual intervention itself. Being able to visually see patterns of alcohol use on our assessment calendar was illuminating and motivating for some, which is consistent with research finding that the simple act of measuring behaviors and comparing them to external standards or goals can result in lasting behavior change [33], with larger effects on goal attainment when outcomes are physically tracked and recorded [34].

Importantly, several participants reacted negatively to the term *intervention*, which was used to describe study treatment conditions in several study-related documents and contact scripts. For these individuals, the term *intervention* triggered negative associations with confrontational Johnson-style interventions [35] during which a person with an addiction is confronted by family and friends and sent to long-term rehabilitation, sometimes against their will. After learning of this unintended association, and the negative reaction across several participants, in July 2020, we removed this term from study documents. For example, in the consent form, we changed references from study *interventions* to study *conditions* or *treatments*. In addition, using prehabilitation program language could also reduce stigma by indicating the program’s goals are to optimize a patient’s health for surgery and subsequent recovery. In our next study phase, we will engage community members in reviewing all our study documents prior to recruitment for clarity and interpretation.

### Limitations and Future Directions

This study has several limitations. Recruitment was paused due to the COVID-19 pandemic and the resulting cancellation of elective surgeries. When surgeries and recruitment resumed at a reduced capacity, it is possible that surgeries and participants were different in significant ways from those scheduled prior to the pandemic. Interviews were conducted by a researcher on the study team, which may have increased the risk of social desirability bias; however, it was a team member who had not been in contact with participants prior to the interview (ie, not the health coach or recruiter), and we reminded participants that the purpose of the interview was to inform intervention improvement, and they were encouraged to provide honest and critical feedback. Finally, while qualitative data can help deepen understanding of participants’ intervention experience, results from this study should not be considered generalizable to broader populations. The next step in research includes a fully powered sequential multiple assignment randomized trial that will randomly assign participants to treatment at baseline and then reassign them to different treatments and or treatment intensities based on initial treatment response and low response.

### Conclusions

In conclusion, through in-depth interviews with a select sample of participants in the ASPIRE pilot trial, we found that both health coaching and brief advice conditions were acceptable pending some minor improvements and modifications. Further research is needed to test the effectiveness of these interventions in reducing drinking before and after surgery, both in the short and long term. Future research and programmatic work on these topics should carefully consider the use of lay versus technical (ie, psychiatric) terminology (eg, *intervention*) to avoid unintended negative reactions. Future research should also investigate whether assessments and feedback alone, without being coupled with brief interventions or health coaching, may be sufficient in changing participant alcohol use behaviors before or after surgery.

## Appendix

Multimedia Appendix 1 Patient qualitative interview guide.

## Abbreviations

ASPIRE: Alcohol Screening and Preoperative Intervention Research
AUDIT-C: Alcohol Use Disorders Identification Test Consumption
TLFB: Timeline Follow Back
