# Supplementary material for: Reducing Alcohol Use Before and After Surgery: Qualitative Study of Two Treatment Approaches
Source: JMIR Perioper Med. 2023 Jul 26;6:e42532. doi: 10.2196/42532 (PMC10413235; doi:10.2196/42532)
Supplement: Multimedia Appendix 1 [file periop_v6i1e42532_app1.docx]

**ASPIRE STUDY QUALITATIVE INTERVIEW GUIDE**

1. Opening Remarks

*The intent of this portion of the agenda is to welcome participants and make them as comfortable as possible by explaining the interview, letting them know what to expect from the experience, and familiarizing them with the facility (rest rooms, etc.).*

1) Purpose of interview:

a) to elicit information about the participant’s opinion of the session, and how it was related or unrelated to any change in alcohol during the time around your surgery

b) to discuss factors (session related or not) that may have increased or decreased drinking during this time period

c) to identify active ingredients or necessary modifications to alcohol screening and brief session practice prior to surgery

2) Housekeeping: rest rooms, time outs, need for smoking breaks (if necessary)

3) Ground rules:

a) respect for all opinions, We’re interested in hearing whatever you have to say, whether it’s positive or negative

b) contributions are voluntary: Please feel free to express your opinions and share your ideas. If you want to skip a question just let me know.

c) confidentiality: As with all the previous study activities you took part of we will maintain complete confidentiality. The information you share will not be shared outside of our study.

4) Audio recording:

a) Recording is kept private and safe [explain site’s data storage procedures, as necessary]

b) when they are transcribed, participants will be identified by a code with all identifiers removed

1. anonymous quotations may go into reports and publications
   1. **Introductions**

*Here, the facilitator introduces herself/himself and explains her role in the study. Participants has any remaining questions are answered.*

1) Introduce self

2) Introduction of participant: ask what they would like to be called (e.g. if William, may go by Bill).

3) Questions?

**Part 1. DESCRIPTION OF STUDY AND SESSION PROCESS**

You participated in the following study activities, can you confirm that this is accurate?

## YOUR STUDY PARTICIPATION

## {Instructions to interviewer: Cross out or grey out the steps that patient did NOT complete. KEEP FLOW CHART OUT FOR PATIENT TO REFER BACK TO}

OR

**Brief Advice**

Study randomization (like a coin flip)

Consent and baseline survey

Defining Terms: Session refers to the health coaching or brief advice sessions you took part in

**Health Coaching Session 2**

4 month Follow up

(post-op)

**Surgery**

Urine and Blood Sample

1 month

Follow up Survey

1 week before

**Health Coaching Session 1**

1. LEAD QUESTION: Why did you chose to participate in this study?
   1. Probes: To reduce use, to benefit self, to benefit others, money, intellectual interest??
2. Thinking back to when you first heard about the study- What did you think about it?
   1. As a topic of research
   2. As something you could participate in
3. LEAD QUESTION: What was participation in this study like for you?
   1. Did it meet your expectations?
   2. What stood out to you?
   3. Highlights?
   4. Lowlights?
4. What were the biggest challenges you faced with regards to participating in this study before surgery?
   1. How about the biggest challenges completing surveys?
5. How was this session different than other information or healthcare you have received in the past related to alcohol use or surgical health?
   1. If this is first alcohol session/information, what was it like receiving this information for the first time?
   2. Was any of the information surprising?

**Part 2: DESCRIPTION OF SESSION OUTCOME**

1. LEAD QUESTION: What change to your alcohol use took place, if any, as a result of study participation?

CHOOSE A, B, or C below.

- 1. Based on your survey responses you submitted to us, it looks like you reduced your alcohol use both before and after surgery, do you have a sense of why your behavior changed in that way?
  2. Based on your survey responses you submitted to us, it looks like your alcohol use remained pretty steady, do you have a sense of why your behavior stayed the same?
  3. Based on your survey responses you submitted to us, it looks like your alcohol use changed before your surgery but went back up afterwards, do you have a sense of why your behavior changed in that way?

1. In what ways did participation impact your alcohol use (if at all)?
   1. Probes
      1. Changed your Thoughts
      2. Changed your Feelings
      3. Changed your Behaviors
      4. Led to conversation with others
      5. Led to an insight or ‘a-ha’ moment
2. We went through what all being a part of this study involved, thinking about that- What aspects of study participation influenced your alcohol use?
3. How did participation in the session (that meeting or phone call you had with our health coach) impact other aspects of your life?
   1. Drug use
   2. Smoking
   3. Stress
   4. Conversations with your doctor
   5. Experience as a patient
4. In what ways did participation in the session influence your decisions related to alcohol use AFTER surgery
   1. In terms of when you first used alcohol again after surgery
   2. In terms of whether you used opioid-based pain medication
   3. Do you have a sense for how having a Major vs minor surgery impacted your decision about when to drink again after surgery?

**Part 3. EVALUATION OF SESSION PROCESS**

1. LEAD QUESTION: What components of the session were helpful?
   1. How could it be improved?
   2. Some of the things we’ve heard from other people are
      1. Weekly interactive check ins before and after their surgery,
      2. Summary notes after the session is complete.
      3. More presentation of scientific research on surgical complications related to alcohol use either in the session handouts or available for you to look at after the session
         1. How much do you agree with these? Why? What else would you add?”
2. What components of the session were undesirable?
3. As part of the session, you were given written information (that packet or infographic that was emailed to you ahead of time), how influential was this information?
4. What did the person delivering the session do that was helpful?
5. One of the suggestions we’ve heard is to have another meeting with the health coach. How do you think having another meeting with the health coach after your surgery would be helpful?
   1. What would you like that meeting to include?
   2. When would you like it happen (phone, video, chat message)? How long would you like it to be? How many times?
   3. How in depth/interactive? (more info, more of a discussion)
6. If you could change one thing about the session what would it be?

HEALTH COACHING ONLY

1. As part of the health coaching condition you created a plan for change including things to do and things to avoid, how influential was this process in changing your alcohol use?
2. The health coach was trained to engage you in a discussion about your alcohol use and surgery, and elicit your thoughts, opinions, and goals, what was it like engaging in this process
   1. What insights about alcohol use or health did you have as a result of discussion?
   2. How did the discussion impact your alcohol use (if at all)?

**Part 4. EVALUATION OF MODALITY AND DELIVERY**

1. LEAD QUESTION: What was your impression of the person delivering the session?
   1. Expertise
   2. Ability to talk with
   3. Ability to help you feel comfortable/at ease
   4. Empathy/Understanding
2. How big a factor was this person in your experience of the session (positive or negative)
3. What ‘type’ of provider would you *ideally* like to deliver this session?
   - 1. Surgeon
     2. Nurse
     3. Social worker
4. Would you prefer this session take place during a pre-existing clinical visit or appointment?
   1. Which one? Pre-op at Domino’s farms, surgeon visit, etc.
5. How was it taking part in the session in person (pre-COVID only)/by phone/video call?
   1. How did the delivery method impact your participation or engagement?
   2. In person wasn’t an option for you due to the impact of COVID, but if you had been able to do so would you have preferred it to the phone/video call?
   3. Did you have any specific challenges with the online/phone format?
6. How do you prefer getting health information or programs like this in light of COVID?
7. If you were to take part in this study again what aspects of session delivery would you ask us to change and why?

**Part 5. Timing of Change related to Surgery**

1. LEAD QUESTION: What other factors outside the study impacted your alcohol use in the past 4 months?
   1. How did preparing for surgery impact alcohol use?
   2. How did the type of surgery (e.g. major or minor) impact your alcohol use?
   3. How did conversations with your healthcare providers impact alcohol use?
   4. How did recovery from surgery impact alcohol use?
   5. How did pain impact your alcohol use or your other drug use?
   6. How did having a prescribed opioid impact your alcohol use (if you had one)?
   7. How did the COVID pandemic impact your alcohol use? Substance use?
2. At the session, you indicated your motivation to change was an X out of 10, and confidence was Y out of 10, how did your motivation and confidence change after the session was over?
   1. How did it change as your surgery date approached?
   2. How did it change after surgery?
3. What were the biggest challenges you faced with regards to changing alcohol use before surgery?
   1. How about the biggest challenges after surgery?

**Part 6. Accuracy in reporting**

1. TLFB: In the surveys you completed for our study, you were asked to fill in a calendar where you reported how much alcohol and/or opioid based pain medications you used each day. These next questions ask about your experience filling out the calendar.
   1. We know it can be difficult to remember/estimate your alcohol use sometimes. How accurate do you think you were able to be when filling in the calendar?
   2. How easy was it fill out the calendar for days you drank and/or used opioid based pain medications?
   3. What, if anything, you would change about this survey tool specifically?
2. In the surveys, we asked you to tell us about your drinking patterns and other sensitive topics. I know these topics can be difficult to talk about with other people. Something we’re interested to find out is how comfortable you are sharing accurate information with our study?
   1. PROBE: How comfortable were you sharing accurate information with our health coach (Brooke)? On the online assessments?
   2. Were there some topics that were more difficult to be completely accurate about?
   3. How would your comfort or reporting accuracy change if you were being asked about alcohol or drug use by a healthcare provider?

**Part 7. WRAP UP**

1. What other information seems relevant to share that I have not asked you about today?

*Thank you for your time and for participating in our study.*
